# EIF2B4 promotes hepatocellular carcinoma progression and immune evasion by driving STAT3 translation via a GEF-dependent mechanism

**DOI:** 10.1007/s13402-025-01117-x

**Published:** 2025-10-27

**Authors:** Yirui He, Yunhe Li, Yayi Chen, Sha Liu, Jia Liu, Rui Wei, Jiapeng Zhang

**Affiliations:** 1 Department of Hematology, Department of Urology and Institute of Urology, Department of Pulmonary and Critical Care Medicine, West China Hospital, Sichuan University, Chengdu, China; 2 Department of Thoracic and Cardiac Surgery, Second Affiliated Hospital of Chongqing Medical University, Chongqing, China; 3 Department of Geriatrics, Sichuan Second Hospital of Traditional Chinese Medicine, Chengdu, China

**Keywords:** EIF2B4, Hepatocellular carcinoma, STAT3 signaling, Translational regulation, Tumor immune evasion

## Abstract

**Background:**

Eukaryotic translation regulators have emerged as pivotal modulators of cancer progression and immune evasion. However, their mechanistic contributions in hepatocellular carcinoma (HCC) remain poorly understood. EIF2B4, the δ-subunit of the eukaryotic initiation factor 2B (eIF2B) complex, has not been previously characterized in HCC.

**Methods:**

EIF2B4 expression was analyzed using public datasets and validated in clinical HCC samples. Functional assays, including gain- and loss-of-function experiments, were performed to assess its impact on cell proliferation, apoptosis, migration, and the cell cycle. RNA immunoprecipitation (RIP), luciferase reporter assays, immunoblotting, and mutational rescue were employed to elucidate EIF2B4-mediated translational regulation of STAT3. In vivo mouse models and immune co-culture systems were used to investigate the role of EIF2B4 in antitumor immunity and response to anti-PD-1 therapy.

**Results:**

EIF2B4 was significantly upregulated in HCC and associated with poor prognosis. EIF2B4 promoted oncogenic phenotypes, including proliferation, migration, and cell cycle progression, while suppressing apoptosis. Mechanistically, EIF2B4 enhanced STAT3 protein expression by directly binding its mRNA and facilitating translation without affecting mRNA levels. EIF2B4 interacted with the eIF2 complex and required GEF activity to promote STAT3 translation via the 5′ untranslated region (5′UTR). GEF-inactivating mutations abolished EIF2B4’s translational and tumor-promoting effects. In vivo, EIF2B4 impaired CD8^+^ T cell-mediated cytotoxicity, reduced immune infiltration, and diminished the efficacy of anti-PD-1 therapy. Conversely, EIF2B4 knockout restored antitumor immunity and sensitized tumors to immune checkpoint blockade.

**Conclusions:**

EIF2B4 functions as a previously unrecognized translational regulator that promotes HCC progression and immune evasion by enhancing STAT3 translation through a GEF-dependent mechanism. These findings highlight EIF2B4 as a potential therapeutic target and biomarker to improve immunotherapy responsiveness in HCC.

**Clinical trial number:**

Not applicable.

**Supplementary Information:**

The online version contains supplementary material available at 10.1007/s13402-025-01117-x.

## Introduction

Hepatocellular carcinoma, the third leading cause of cancer-related mortality worldwide, continues to pose significant therapeutic challenges [1, 2]. While immune checkpoint blockade (ICB) has emerged as a promising strategy for advanced-stage patients, the objective response rate remains below 30%, suggesting the presence of undiscovered resistance hubs within the tumor microenvironment (TME) [3–5]. Recent studies have revealed that dysregulated translational control in cancer cells not only drives malignant proliferation but also remodels the immune microenvironment through secretory factors [6–8]. However, the core molecular mechanisms mediating this bidirectional regulation remain poorly defined.

Eukaryotic translation initiation factor 2B (eIF2B), a rate-limiting hub for protein synthesis, catalyzes the regeneration of eIF2-GTP to govern global translation rates [9, 10]. Notably, its epsilon subunit (EIF2B4) exhibits tumor-specific overexpression across multiple solid malignancies compared to other eIF2B subunits [11], yet its canonical role in translation initiation and functional role in HCC progression remains systematically unexplored [12]. Through bioinformatic analyses, we identified significant upregulation of EIF2B4 in HCC tissues, which correlated closely with poor patient prognosis. Functional enrichment analyses further suggested that EIF2B4 may drive HCC progression via dual mechanisms: directly accelerating tumor cell cycle progression through translation reprogramming, and shaping an immunosuppressive microenvironment via aberrant synthesis of immune-modulatory molecules.

In this study, we systematically elucidate the oncogenic function and immunoregulatory role of EIF2B4 in HCC. Mechanistically, EIF2B4 facilitates the translation of STAT3 mRNA, sustaining the expression of STAT3 protein and its downstream targets to promote cell cycle progression and inhibit apoptosis. Further mechanistic investigations revealed that EIF2B4 interacts with the eIF2 complex and promotes STAT3 translation in a GEF activity-dependent manner, pinpointing EIF2B4 as a functional mediator of oncogenic mRNA translation initiation. The tumor-promoting effect of EIF2B4 was found to be STAT3-dependent, as STAT3 knockout completely abolished EIF2B4-driven phenotypes. In addition, EIF2B4 impairs antitumor immunity by reducing tumor susceptibility to CD8+ T cell-mediated cytotoxicity. Importantly, genetic ablation of EIF2B4 enhances the efficacy of anti-PD-1 therapy in vivo, accompanied by reduced PD-L1 expression and increased immune infiltration. These findings identify EIF2B4 as a critical translational regulator that bridges oncogenic signaling with immune escape, and highlight its therapeutic potential as a combinatorial target to improve ICB responsiveness in HCC.

## Methods and materials

### Identification of gene expression profiles

The pan-cancer expression profile of EIF2B4 was retrieved from the TIMER2.0 database (http://timer.cistrome.org/). RNA-seq data (HTSeq-FPKM format) and clinical annotations for HCC patients were obtained from the TCGA-LIHC cohort via the UCSC Xena Browser (https://xenabrowser.net). FPKM values were converted to transcripts per million (TPM) using custom R scripts (v4.10). Differential expression analysis of EIF2B4 in HCC tumor versus adjacent normal tissues was performed using the limma package, with significance thresholds set at |log2(fold change) | ≥1 and adjusted *p* < 0.05. Volcano plots and expression boxplots were visualized using ggpubr. For subgroup analyses, HCC patients were stratified into high/low EIF2B4 expression groups (top/bottom 30% by TPM), and differentially expressed genes (DEGs) were identified under the same thresholds. Heatmaps of DEGs were generated using pheatmap. Functional enrichment analysis of DEGs for Gene Ontology (GO) terms and KEGG pathways was conducted using the DAVID database (v6.8, https://david.ncifcrf.gov), with results visualized via ggplot2 (v3.4.2) and ggpubr.

### Clinical prognostic evaluation

The prognostic significance of EIF2B4 in HCC was assessed using R packages survival (v3.5–5), survminer (v0.4.9), and timeROC (v0.4). Kaplan-Meier (KM) survival curves with log-rank tests and time-dependent receiver operating characteristic (ROC) curves (1-/3-/5-year overall survival) were constructed. A nomogram integrating EIF2B4 expression and clinical parameters (age, TNM stage, grade) was developed using rms (v6.7–0) and regplot (v1.1), with calibration curves evaluating prediction accuracy. Univariate and multivariate Cox proportional hazards regression analyses were performed to identify independent prognostic factors, visualized as forest plots using survminer.

### Impact of EIF2B4 on tumor immune function

The TME composition was deconvoluted using the ESTIMATE algorithm (estimate package, v1.0.13) to calculate stromal/immune scores. Correlation analyses between EIF2B4 expression and tumor mutation burden (TMB) or immune checkpoint genes (CD44, CD27, LAG3) were performed using limma, with results visualized via ggplot2, ggpubr, and corrplot (v0.92). Spearman’s rank correlation coefficients were computed to assess associations, and all plots were refined for clarity using ggExtra (v0.10.0). Statistical significance was defined as two-tailed *p* < 0.05.

### Cell culture

The 293T, L02, Huh7, Hep3B, and Hepa1–6 cell lines (purchased from ATCC) were cultured in DMEM or RPMI-1640 medium (Gibco) supplemented with 10% fetal bovine serum (FBS, Gibco) at 37 °C under 5% CO₂.

### Gene editing

Genetic knockout and overexpression cell line models of EIF2B4 and STAT3 were generated via lentiviral transfection. Gene-specific sgRNAs targeting EIF2B4/Eif2b4 were cloned into the lentiCRISPRv2 vector (Addgene). Cas9-expressing cells were transduced with lentiviral particles produced by co-transfecting 293T cells with packaging plasmids (psPAX2 and pMD2.G) using the calcium phosphate method. For gene overexpression, the full-length cDNA was amplified by PCR and cloned into the pcDNA3.1 vector via homologous recombination. Lentiviral supernatants were harvested at 36 h and 48 h post-transfection, and used to transduce target cells (70% confluency) with 8 μg/mL polybrene. Transduced cells were centrifuged at 2,000×g for 60 min at 37 °C and selected with puromycin (2 μg/mL).

### RNA sequencing

Total RNA was extracted from triplicate biological replicates of WT and EIF2B4-KO Hep3B cells using TRIzol (Invitrogen). Libraries were prepared using the TruSeq Stranded mRNA Kit (Illumina) and sequenced on a NovaSeq 6000 platform. Raw reads were aligned to the human genome (GRCh38) using STAR (v2.7.10a), and DEGs were identified with DESeq2 (v1.38.3; |log2FC| ≥1, FDR < 0.05). Functional enrichment analysis was performed using clusterProfiler (v4.8.1).

### Animal models

Six- to eight-week-old male C57BL/6 mice (GemPharmatech LLC) were subcutaneously inoculated with 3 × 10^6^ Hepa1–6 cells (WT or Eif2b4-KO) in 50 μL PBS. Tumor volumes were calculated as V = (L × W^2^)/2, where L (length) and W (width) were measured using digital calipers. Anti-PD-1 therapy (5 mg/kg, Selleck, #A2122) was administered intraperitoneally twice weekly starting at day 7 post-inoculation. Body weight and tumor size were monitored every 3 days.

### In vitro malignant phenotyping

Migration Assay: Cells (2 × 10^4^) in serum-free medium were seeded into Transwell inserts (8 μm, Corning). Complete medium with 10% FBS was added to the lower chamber. After 24 h, migrated cells were fixed with 4% paraformaldehyde, stained with 0.1% crystal violet, and imaged under an inverted microscope.

Cytotoxicity Assay: CD8^+^ T cells (isolated from healthy donor PBMCs using magnetic beads) were co-cultured with tumor cells (5:1 ratio) for 48 h. Cells were stained with Calcein AM (1 μM) and propidium iodide (PI, 5 μg/mL) for 30 min, and viability was quantified via fluorescence microscopy.

### Flow cytometry

Apoptosis: Cells (1–5 × 10^6^/mL) were stained with Annexin V-FITC/PI (Beyotime) in binding buffer and analyzed on a BD Accuri C6. Gates were defined using unstained and single-stained controls.

Cell Cycle: Ethanol-fixed cells (70%, 4 °C, 30 min) were treated with RNaseA (100 μg/mL) and PI (50 μg/mL). DNA content was analyzed based on forward/side scatter (FSC/SSC) profiles using FlowJo (v10.4.0).

### STAT3 RNA immunoprecipitation

RNA-binding activity of EIF2B4 was evaluated using Magna RIP™ RNA-Binding Protein Immunoprecipitation Kit (Millipore) following the manufacturer’s protocol [13, 14]. Briefly, Hep3B cells were lysed in RIP lysis buffer with RNase inhibitors and protease inhibitors. Cell lysates (2 × 10^7^ cells per IP) were incubated with magnetic beads pre-bound with either normal IgG (negative control), anti-EIF2B4 (Proteintech), or anti-FLAG (Sigma) antibodies overnight at 4 °C. After extensive washing, RNA-protein complexes were digested with Proteinase K and immunoprecipitated RNA was purified using phenol–chloroform extraction. Reverse transcription was performed with random primers, and qPCR was conducted to quantify STAT3 mRNA enrichment using input RNA as reference. Four groups were included (1): WT, immunoprecipitated with anti-EIF2B4; (2) EIF2B4-KO, anti-EIF2B4; (3) FLAG-EIF2B4 rescue, anti-FLAG; and (4) IgG control. Relative enrichment was calculated as %input using the ΔCt method.

### Statistical analysis

All quantitative data are presented as mean ± SD (technical replicates) or mean ± SEM (biological replicates). Normality was assessed using the Shapiro-Wilk test. Between-group differences were analyzed by two-tailed Student’s t-test (parametric) or Mann-Whitney U test (non-parametric). Survival analyses employed Kaplan-Meier curves with log-rank testing. Correlations between continuous variables were evaluated using Pearson’s (normal distribution) or Spearman’s (non-normal) coefficients. Transcriptomic differential expression thresholds were set at FDR < 0.05 (Benjamini-Hochberg correction). Effect sizes are reported as Cohen’s d (for t-tests) or hazard ratios (for survival analyses). All analyses were performed using GraphPad Prism 9.0 and R 4.2.1, with statistical significance defined as *p* < 0.05.

## Results

### EIF2B4 overexpression predicts poor survival and serves as an Independent prognostic biomarker in HCC

**Fig. 1:**
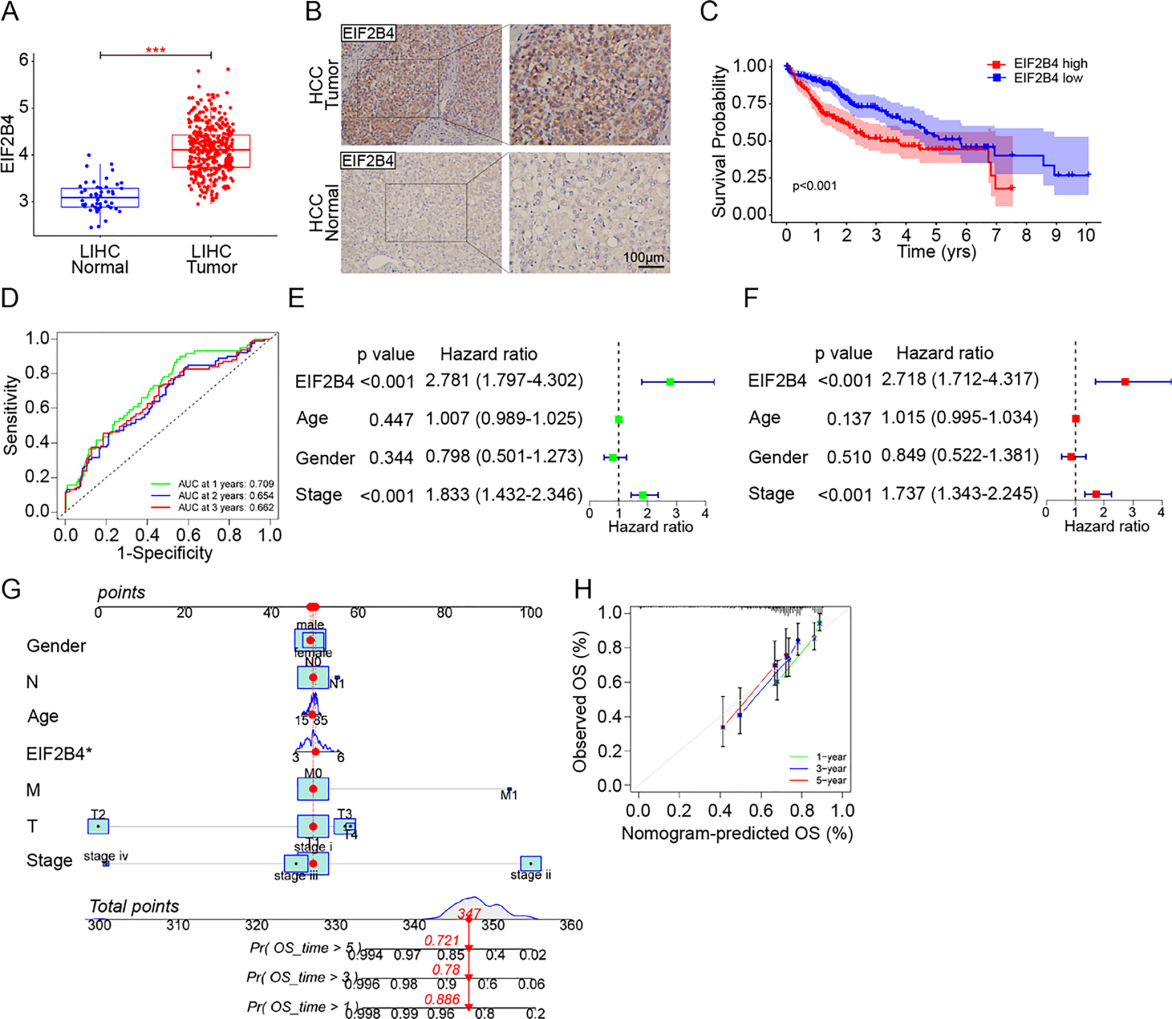
EIF2B4 is Overexpressed in HCC and Predicts Poor Prognosis. (**A**). Elevated EIF2B4 mRNA expression in LIHC tumors compared to adjacent normal tissues (TCGA data). (**B**). Representative immunohistochemical (IHC) staining of EIF2B4 in HCC and paired normal tissues (Scale bar = 100 µm). (**C**). Kaplan-Meier survival curves showing reduced overall survival in HCC patients with high EIF2B4 expression (log-rank *p* < 0.001). (**D**). Time-dependent ROC curves demonstrating the prognostic accuracy of EIF2B4 for 1-, 3-, and 5-year survival (AUC values indicated). (**E-F**). Forest plots of univariate (**E**) and multivariate (**F**) Cox regression analyses identifying EIF2B4 as an independent prognostic factor. (**G-H**). Nomogram (**G**) and calibration curve (**H**) integrating EIF2B4 expression with clinical parameters for survival prediction

Integrated analysis of TCGA pan-cancer data revealed significant upregulation of EIF2B4 in LIHC tissues compared to adjacent normal controls (*p* < 0.001, Supplementary fig. 1, Fig. 1A–B). Survival profiling demonstrated that patients with high EIF2B4 expression exhibited markedly shorter overall survival (OS) (Fig. 1C). Time-dependent ROC analysis further established the stable prognostic power of EIF2B4 expression, with area under the curve (AUC) values of 0.709, 0.654, and 0.662 for 1-, 3-, and 5-year survival prediction, respectively (Fig. 1D). Univariate (HR = 2.781, 95%CI 1.797–4.302, *p* < 0.001, Fig. 1E) and multivariate (HR = 2.718, 95%CI 1.712–4.317, *p* < 0.001, Fig. 1F) cox regression analyses confirmed EIF2B4 as an independent prognostic factor for HCC patients. The predictive accuracy was visually confirmed through a nomogram model (Fig. 1G) and calibration curve (Fig. 1H)

### Functional enrichment analysis implicates EIF2B4 in cell cycle regulation in HCC

**Fig. 2:**
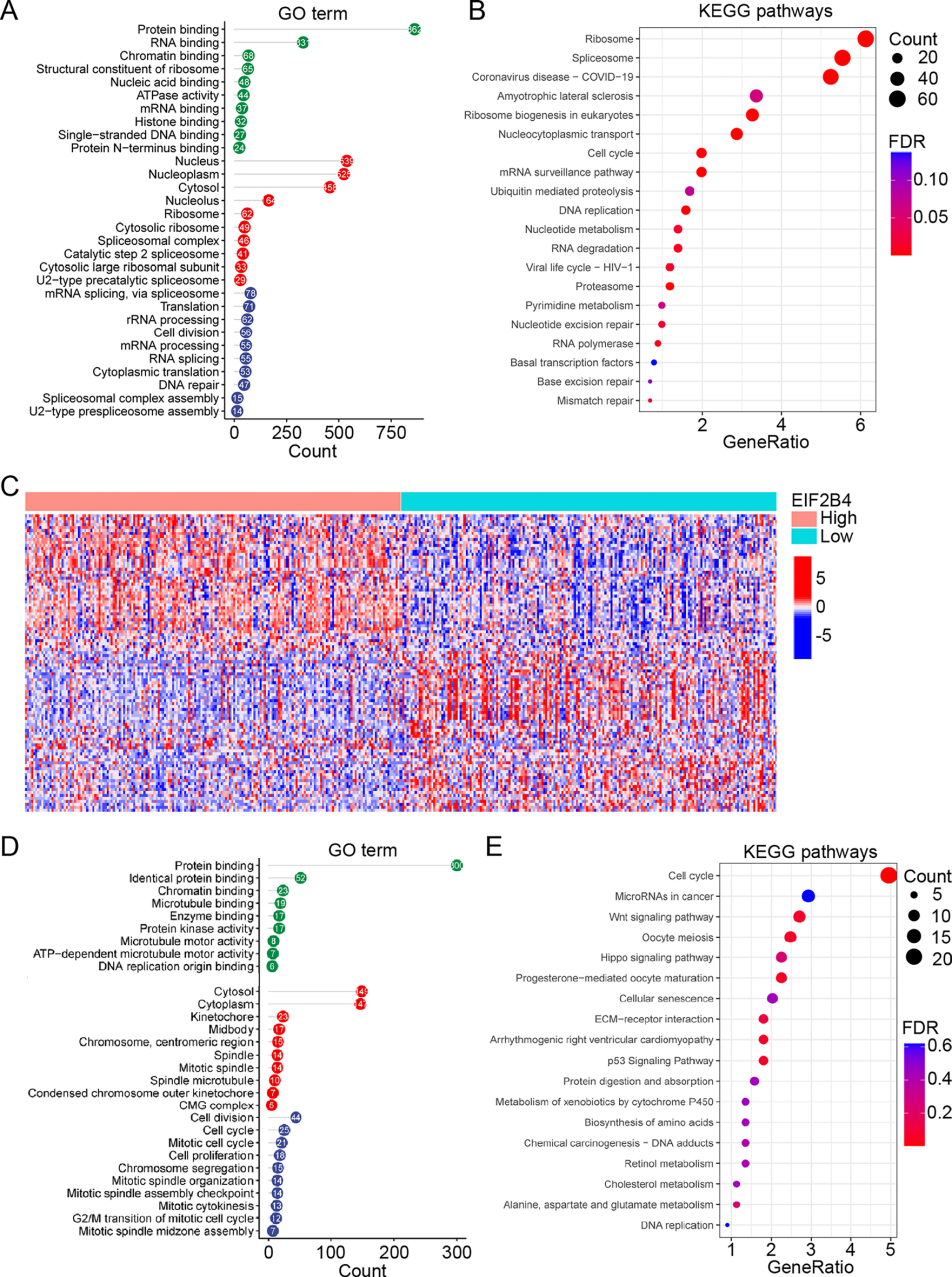
Functional Enrichment of EIF2B4 Co-expressed Genes in HCC. (**A-B**). GO biological processes (**A**) and KEGG pathways (**B**) enriched among genes co-expressed with EIF2B4 in HCC. (**C**). Heatmap of top DEGs stratified by EIF2B4 expression levels. (**D-E**). GO (**D**) and KEGG (**E**) pathway analyses of DEGs in high vs. low EIF2B4 expression groups

Functional enrichment analysis of genes co-expressed with EIF2B4 in HCC patient samples revealed significant enrichment in biological processes related to translation, cell division, and mRNA splicing/processing (Fig. 2A). KEGG pathway analysis further showed associations with ribosome function, spliceosome activity, cell cycle progression, and DNA replication (Fig. 2B), suggesting a potential role of EIF2B4 in coordinating cell cycle dynamics through translational regulation.

To further investigate EIF2B4’s biological functions, HCC patients were stratified into high- and low-expression groups. Heatmap visualization of the top 100 differentially expressed genes demonstrated distinct transcriptional profiles between groups (Fig. 2C). Subsequent enrichment analysis indicated that upregulated genes in the high-EIF2B4 group were primarily involved in cell division, cell cycle progression, and proliferative processes (Fig. 2D). KEGG pathway analysis corroborated these findings, with significant enrichment in cell cycle-related pathways (Fig. 2E). These results collectively suggest that EIF2B4 may promote HCC progression through cell cycle regulation, providing a theoretical basis for mechanistic studies.

### EIF2B4 drives hepatocellular carcinoma progression by remodeling the tumor microenvironment and genomic instability

The functional enrichment analyses preliminarily revealed that EIF2B4 promotes tumor progression through translational regulation and cell cycle control. However, tumor progression depends not only on cell-autonomous growth but also on microenvironmental remodeling and genomic evolution [15–17]. To investigate whether EIF2B4 synergistically drives malignant phenotypes via non-cell-autonomous mechanisms, we systematically evaluated its impact on TME composition, genomic stability, and immune checkpoint regulation.

**Fig. 3:**
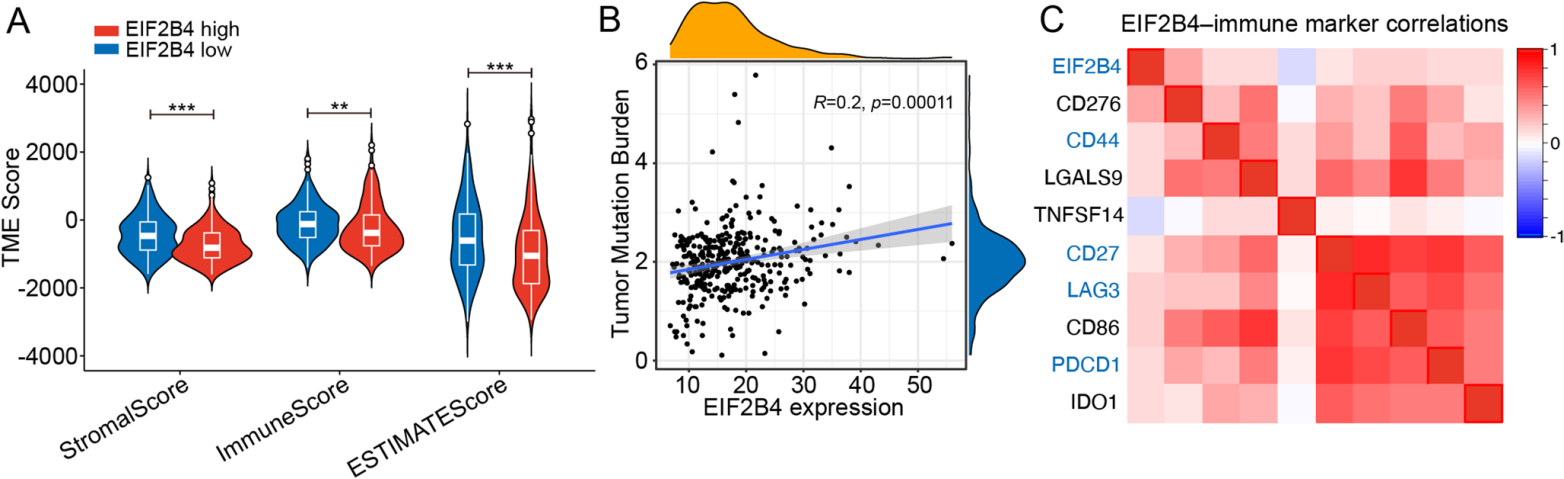
EIF2B4 Modulates Tumor Microenvironment and Genomic Instability. (**A**). ESTIMATE algorithm-derived stromal/immune scores in EIF2B4-high vs. low HCC tumors. (**B**). Positive correlation between EIF2B4 expression and tumor mutation burden (TMB) (*R* = 0.2, *p* < 0.001). (**C**). Association of EIF2B4 expression with immune checkpoint markers

Using the ESTIMATE algorithm [18], we first assessed the influence of EIF2B4 on stromal and immune components in HCC. The results demonstrated that elevated EIF2B4 expression significantly suppressed the infiltration and proportion of stromal and immune cells within the TME (Fig. 3A). Furthermore, TMB, a hallmark of malignancy [19], exhibited a positive correlation with EIF2B4 levels (Fig. 3B), suggesting that EIF2B4 may exacerbate genomic instability to fuel tumor progression. To evaluate the clinical implications of EIF2B4-mediated immune microenvironment alterations, we analyzed its correlation with key immune checkpoint molecules. Notably, EIF2B4 expression positively correlated with PDCD1, LAG3, CD27, and CD44 (Fig. 3C), indicating its potential role in inducing immune resistance and impairing clinical responses to ICB [20]. These findings collectively provide a theoretical foundation for targeting EIF2B4 to overcome therapeutic resistance.

### EIF2B4 drives tumor cell growth in HCC via pro-proliferative and anti-apoptotic mechanisms

**Fig. 4:**
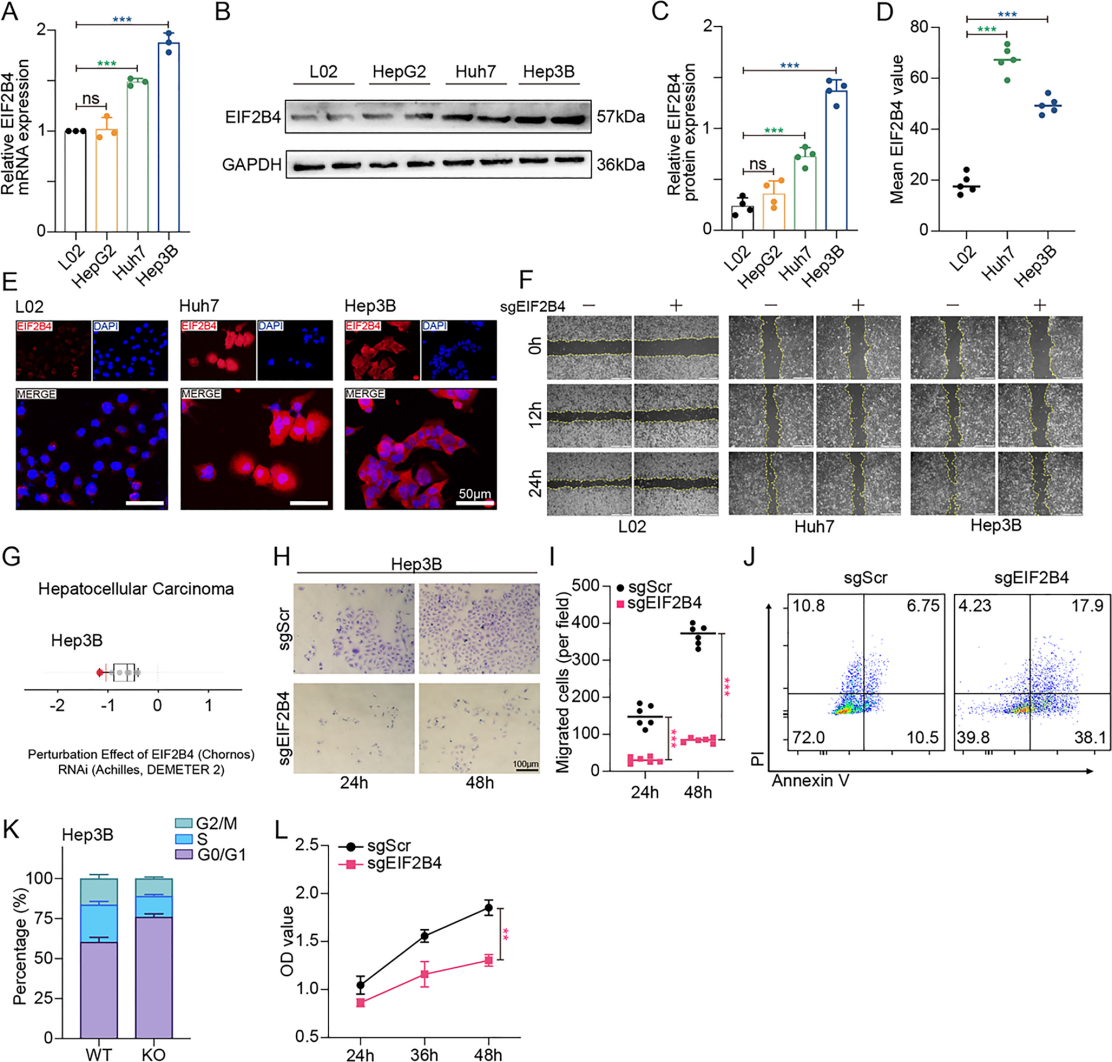
EIF2B4 Dependency and Functional Validation in HCC Cell lines. (**A**). qPCR validation of EIF2B4 mRNA levels in HCC cell lines (Hep3B, Huh7 and HepG2) vs. normal hepatocytes (L02). (**B-C**). Representative Western blot (**B**) and quantification (**C**) of EIF2B4 protein expression among cell lines mentioned in Fig. 4A. (**D-E**). Immunofluorescence staining (**E**) and intensity quantification (**D**) of EIF2B4 (Scale bar = 50 µm). (**F**). Time-lapse images of scratch wounds in L02, Huh7, and Hep3B cells pre- and post-EIF2B4 knockout (0 h, 12 h and 24 h). (**G**). Chronos dependency scores of EIF2B4 across human HCC cell lines (CCLE database). (**H-I**). Representative bright-field images (**H**) and quantification (**I**) of migratied Hep3B cells (WT and EIF2B4-KO). (**J-K**). Flow cytometric analysis of apoptosis (**J**) and cell cycle distribution (**K**) in EIF2B4-KO Hep3B cells. (**L**). Quantitative analysis of cell proliferation by CCK-8 demonstrating attenuated growth of Hep3B post EIF2B4 KO

To delineate the functional impact of EIF2B4 on HCC tumorigenesis, we first quantified its expression across HCC cell lines. Consistent with prior findings, HepG2, Huh7, and Hep3B cells exhibited significantly higher EIF2B4 expression at both transcriptional and translational levels compared to the normal hepatocyte line L02 (Fig. 4A–E).

EIF2B4 knockout models were established in L02, Huh7, and Hep3B cell lines (Supplementary fig. 1B). Wound healing assays revealed that EIF2B4 depletion markedly impaired the migratory capacity of HCC cells compared to the non-tumorigenic L02 cells (Fig. 4F). Based on Chronos dependency scores, Hep3B was identified as the most EIF2B4-dependent HCC cell line and was therefore selected for subsequent functional investigations (Fig. 4G) [21].

Following evidences showed that, EIF2B4 knockout led to a marked reduction in the proliferation rate and migratory capacity of Hep3B cells in vitro (Fig. 4H–J). Flow cytometric analysis further revealed a significant increase in apoptosis following EIF2B4 depletion. These impaired malignant phenotypes induced by EIF2B4 loss were likely associated with a pronounced cell cycle arrest (Fig. 4K, L), which aligned with bioinformatic predictions that EIF2B4 fuels tumor progression by accelerating proliferation through its canonical translational role [22].

### Transcriptomic profiling reveals EIF2B4 suppresses JAK-STAT signaling via translational control of STAT3

**Fig. 5:**
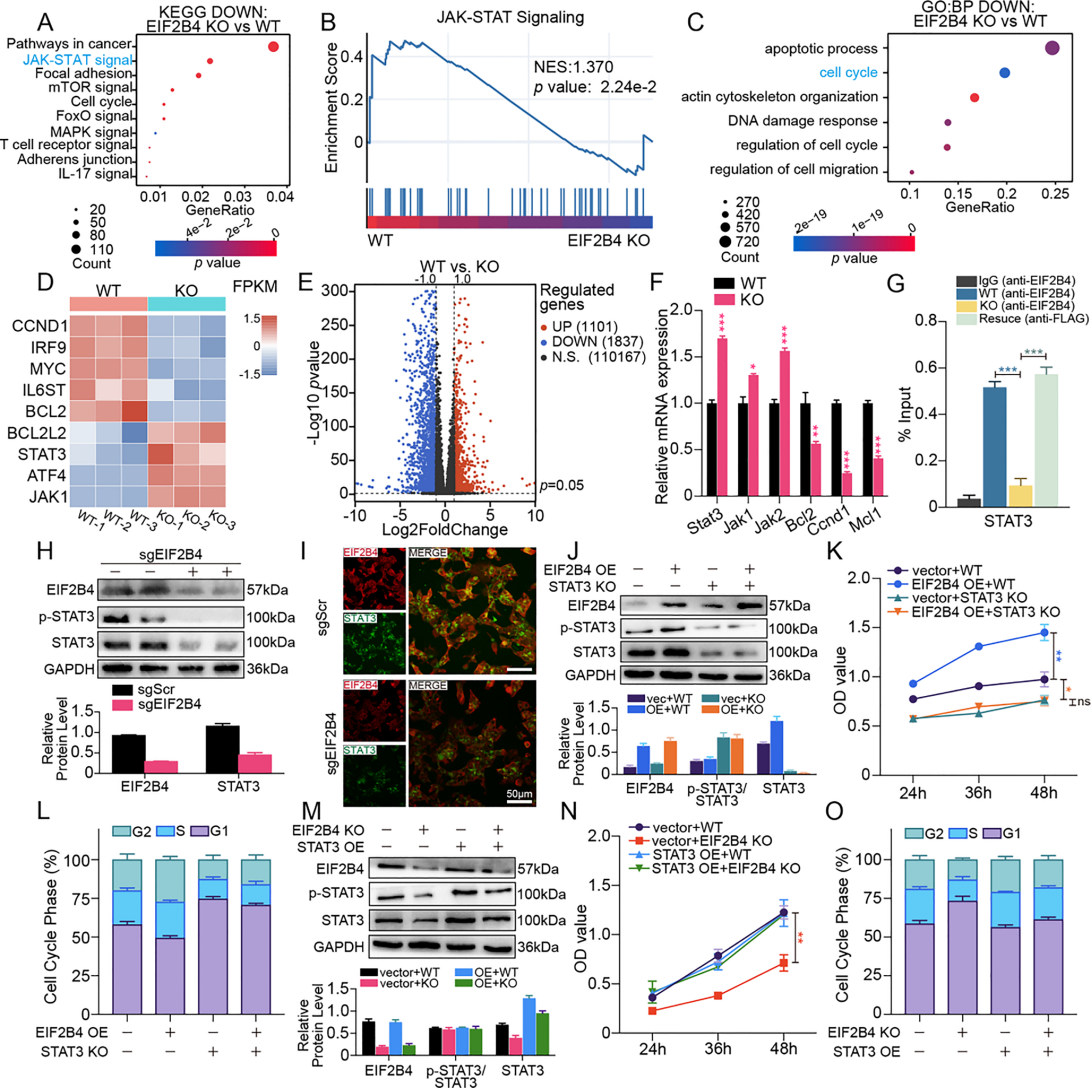
EIF2B4 Regulates JAK-STAT Signaling Through Translational Control of STAT3. (**A**). KEGG enrichment of down-regulated pathways in EIF2B4-KO Hep3B cells. (**B**). GSEA showing suppressed JAK-STAT signaling in EIF2B4-KO cells (NES = 1.370, p < 0.001). (**C**). GO-BP enriched among down-regulated gene signitures. (**D**). Heatmap of JAK-STAT pathway gene expression changes post-EIF2B4 KO. (**E**). Volcano plot of DEGs (|log2FC| > 1, FDR < 0.05). (**F**). qPCR validation of JAK-STAT pathway genes (*: p < 0.05, ** P < 0.01, *** P < 0.001, ns = non-significant). (**G**). RIP-qPCR validation of physical interaction between EIF2B4 and STAT3 mRNA. (**H**). Representative immunoblot and quantitative analysis showing reduced STAT3 and p-STAT3 levels following EIF2B4 KO. (**I**). Representative immunofluorescence images showing decreased STAT3 staining in Hep3B post-EIF2B4 KO. (**J**). Representative immunoblot and quantitative analysis showing increased total STAT3 level by EIF2B4 OE while p-STAT3/STAT3 ratio remain unchanged. (**K**). CCK8 showing that EIF2B4 promotes Hep3B cell proliferation in a STAT3-dependent manner. (**L**). Flow cytometry analysis of cell cycle distribution demonstrates that EIF2B4 accelerates G1-S phase transition in a STAT3-dependent manner. (**M**). Representative immunoblot and quantitative analysis showing selective regulation of total-STAT3 by EIF2B4 without affecting p-STAT3/STAT3 ratio even upon STAT3 rescue. (**N**). CCK8 showing reduced cell proliferation of Hep3B post-EIF2B4 KO, which can be significantly rescued by STAT3 reconstitution. (**O**). Flow cytometry analysis of cell cycle distribution demonstrates that STAT3 OE significantly rescues the G1-S phase arrest induced by EIF2B4 KO

To elucidate the mechanisms underlying EIF2B4-driven tumor proliferation, we performed transcriptomic sequencing on Hep3B cells with or without EIF2B4 knockout (KO). Differential gene expression analysis revealed significant downregulation of the JAK-STAT signaling pathway in KO cells (Fig. 5A, B, Supplementary fig. 1C). GO-BP enrichment analysis indicated that gene signatures related to cell cycle, migration, and apoptosis were concurrently downregulated following EIF2B4 depletion (Fig. 5C), consistent with the phenotypic changes observed in our functional experiments shown in Fig. 4.

Intriguingly, while downstream JAK-STAT effectors (CCND1, MYC, BCL2) were markedly downregulated in KO cells, upstream genes JAK1 and STAT3 exhibited transcriptional upregulation (Fig. 5D–E). The mRNA levels of key molecules in the JAK-STAT signaling pathway were validated by qPCR, showing patterns consistent with transcriptomic data and indicating transcriptional upregulation of upstream genes, including STAT3, upon EIF2B4 depletion (Fig. 5F). However, Western blot analysis revealed that both total and phosphorylated STAT3 protein levels were consistently decreased in EIF2B4-deficient Hep3B cells, although the ratio of phosphorylated to total STAT3 remained unchanged (Fig. 5H). Immunofluorescence further confirmed the downregulation of STAT3 upon EIF2B4 KO (Fig. 5I). This discordance between transcriptional and translational outputs aligns with EIF2B4’s canonical role in translation initiation, suggesting compensatory transcriptional upregulation of STAT3 may fail to offset translational suppression. To further test our hypothesis that EIF2B4 promotes STAT3 expression at the translational level, we performed RNA immunoprecipitation (RIP) assays in Hep3B cells. EIF2B4-specific antibodies were able to significantly enrich STAT3 mRNA in WT cells compared to the IgG control. Such enrichment was markedly attenuated in EIF2B4 KO group. Upon reconstitution of FLAG-EIF2B4 expression, FLAG immunoprecipitation demonstrated restored STAT3 enrichment levels to that observed in WT group, as confirmed by RIP-qPCR (Fig. 5G). Collectively, these results provide mechanistic support for the involvement of EIF2B4 in the translational regulation of STAT3.

To evaluate the dependency of EIF2B4-driven oncogenic phenotypes on STAT3 activity, we constructed recombinant vectors for EIF2B4 overexpression (EIF2B4 OE) and STAT3 knockout (STAT3 KO, Supplementary fig. 1), respectively. EIF2B4 OE led to increased levels of both total and phosphorylated STAT3, while the p-STAT3/t-STAT3 ratio remained stable (Fig. 5J). This was accompanied by enhanced proliferation and accelerated cell cycle progression in Hep3B cells, as indicated by a reduced proportion of cells in G1 phase. In contrast, STAT3 knockout alone significantly impaired tumor cell proliferation and induced G1-phase cell cycle arrest. Notably, reintroduction of EIF2B4 in STAT3-deficient cells failed to restore proliferative capacity or cell cycle progression, indicating that the pro-tumorigenic effects of EIF2B4 are dependent on STAT3 expression (Fig. 5K, L).

We further constructed a STAT3 OE vector to assess its functional rescue capacity. EIF2B4 KO alone effectively reduced both total and phosphorylated STAT3 protein levels (Fig. 5M, Supplementary fig. 1). Functionally, STAT3 OE markedly rescued the proliferation defect and G1-phase cell cycle arrest caused by EIF2B4 depletion in Hep3B cells (Fig. 5N, O), supporting STAT3 as a critical downstream effector mediating EIF2B4-driven oncogenic activity. Reintroduction of STAT3 in EIF2B4-deficient cells significantly restored STAT3 expression, though still slightly lower than that observed in the STAT3 OE group. Notably, the proportion of phosphorylated STAT3 relative to total STAT3 remained comparable between the two groups (Fig. 5M). These protein expression patterns suggest that EIF2B4 may regulate STAT3 at the translational level without substantially affecting its phosphorylation process.

To delineate the functional impact of EIF2B4 on HCC tumorigenesis, we first quantified its expression across HCC cell lines. Consistent with prior findings, HepG2, Huh7, and Hep3B cells exhibited significantly higher EIF2B4 expression at both transcriptional and translational levels compared to the normal hepatocyte line L02 (Fig. 4A–E).

### EIF2B4 knockout sensitizes tumors to immune checkpoint blockade by reversing immunosuppressive microenvironment

**Fig. 6:**
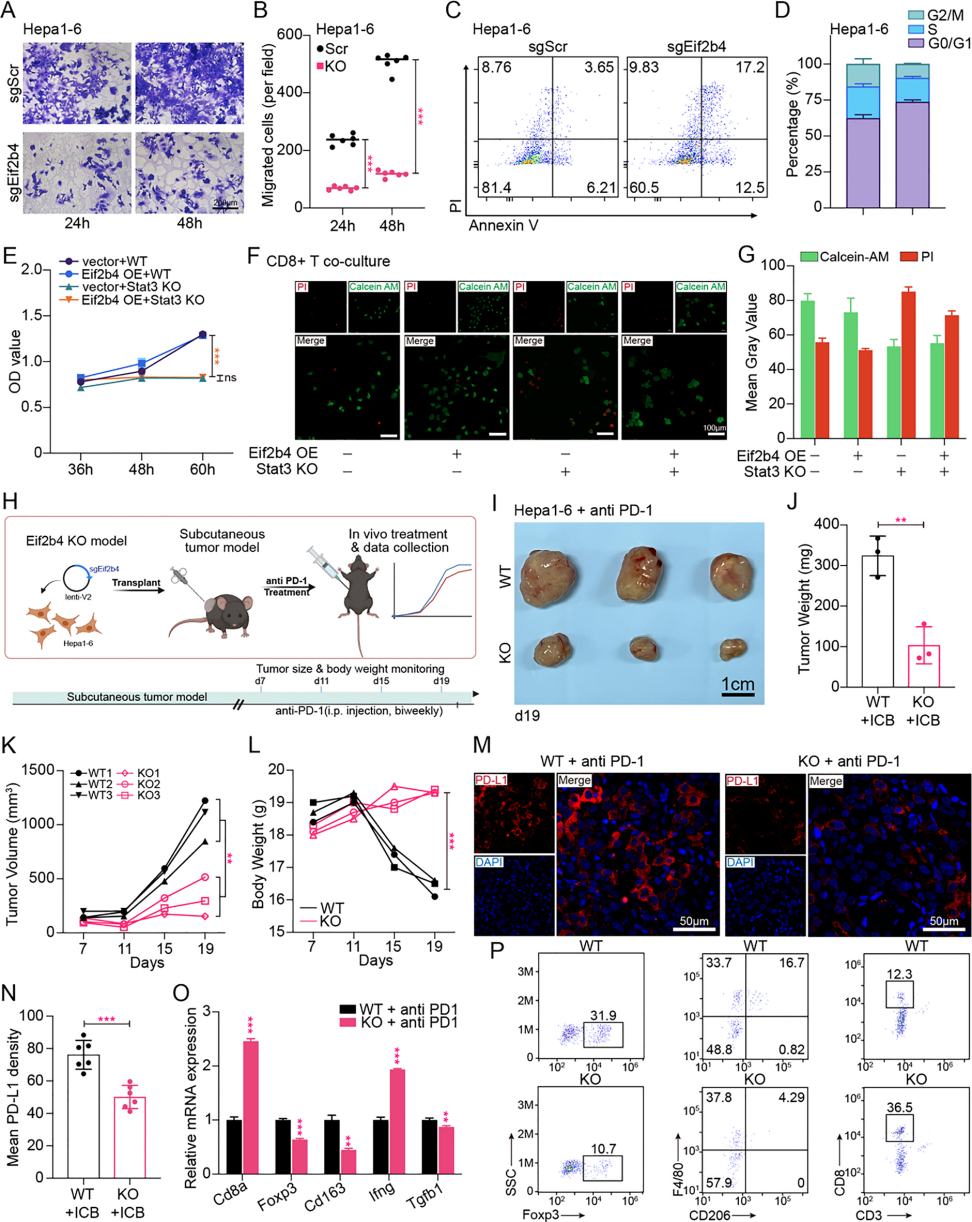
EIF2B4 Knockout Sensitizes HCC to Anti-PD-1 Therapy In Vivo. (**A-B**). Representative bright-field images (**A**) and quantification (**B**) of migrated Hepa1-6 cells (WT and Eif2b4-KO). (**C-D**). Flow cytometric analysis of apoptosis (**C**) and cell cycle distribution (**D**) in Eif2b4-KO Hepa1-6 cells. (**E**). CCK8 showing that Eif2b4 promotes Hepa1-6 cell proliferation in a Stat3-dependent manner. (**F-G**). Representative images (**F**) and quantitative analysis (**G**) showing enhanced CD8+ T cell-mediated killing of Eif2b4-KO tumors and rescue by Stat3-OE (Scale bar = 100 µm). (**H**). Schematic of anti-PD-1 treatment in syngeneic Hepa1-6 tumor models (WT and Eif2b4-KO). (**I-J**). Gross morphology (**I**) and weight (**J**) of subcutaneous tumors at the end of in vivo treatment. (**K-L**). Tumor growth curves (**K**) and body weight changes (**L**) during anti PD-1 treatment. (**M-N**). Representative PD-L1 immunofluorescence (**M**) and quantification (**N**) in tumors from different groups (Scale bar = 50 µm). (**O**). Transcriptional changes of immune markers in Eif2b4-KO vs. WT tumors. (**P**). Flow cytometric analysis showing increased CD8+ T cells and reduced Tregs and M2 macrophages in Eif2b4-KO tumors compared with WT controls

Given that our initial analyses implicated EIF2B4’s function in modulating immune evasion (Fig. 3), we next investigated whether EIF2B4 contributes to immune evasion in HCC. Considering the high evolutionary conservation of EIF2B4 between humans and mice, we first evaluated the oncogenic role of Eif2b4 in the murine HCC cell line Hepa1–6. Consistently, Eif2b4 knockout led to impaired migration, increased apoptosis, and prominent G1 phase arrest in Hepa1–6 cells (Fig. 6A–D, Supplementary fig. 1). Next, the impact of Eif2b4 on immune evasion in HCC was investigated in vitro assays. Under different genetic backgrounds of Eif2b4 OE with or without Stat3 KO, we found that the pro-proliferative effect of Eif2b4 remained dependent on Stat3 (Fig. 6E). Furthermore, co-culture with CD8^+^ T cells demonstrated that the Eif2b4-Stat3 axis modulates the sensitivity of Hepa1–6 cells to cytotoxic T cell–mediated killing (Fig. 6F, G).

Building on in vitro evidence of EIF2B4–STAT3 mediated immune evasion, we investigated the therapeutic impact of EIF2B4 ablation on ICB efficacy in vivo. Using Eif2b4 KO Hepa1–6 cells, we established syngeneic tumors in immune-competent C57BL/6 mice. When tumors reached > 100 mm^3^, mice received anti-PD-1 therapy (5 mg/kg, intraperitoneal administration twice a week). Tumor volume and body weight were monitored every 3 days until reaching the endpoint (maximum volume ~1500 mm^3^) (Fig. 6H).

Anti-PD-1 treatment elicited significantly stronger tumor regression in Eif2b4 KO mice compared to WT controls (Fig. 6I–K). Therapeutic divergence emerged as early as day 15 post-treatment and widened over time. Notably, WT mice exhibited progressive weight loss from day 11 onward, whereas KO mice maintained stable body weight (Fig. 6L). This weight divergence likely reflects differential tumor burden or treatment tolerance conferred by Eif2b4 KO.

Immunohistochemistry of excised tumors confirmed reduced PD-L1 expression intensity and distribution in KO tumors (Fig. 6M–N), corroborating Eif2b4’s role in sustaining immune evasion. Transcriptomic profiling further revealed elevated Cd8a and Ifng (markers of cytotoxic T cell infiltration/activity) alongside reduced Foxp3 (Tregs), Cd163 (M2-polarized tumor-associated macrophages), and Tgfb1 (immunosuppressive cytokine) in KO tumors (Fig. 6O). Consistently, flow cytometry analysis demonstrated a higher proportion of CD8^+^ T cells together with decreased Tregs and M2 macrophages in KO tumors (Fig. 6P). These findings collectively demonstrate that Eif2b4 ablation remodels the immunosuppressive TME by enhancing T cell recruitment/activation while dampening inhibitory immune subsets.

In summary, our data establish that EIF2B4 knockout restores ICB sensitivity through dual mechanisms: direct suppression of PD-L1-dependent adaptive resistance, and rebalancing the immune landscape toward pro-inflammatory states. This work positions EIF2B4 as a master regulator of tumor-immune crosstalk and a promising therapeutic target for combination immunotherapy.

### EIF2B4 exerts translational regulation over STAT3 via its interaction with the eIF2 complex and 5′UTR binding

**Fig. 7:**
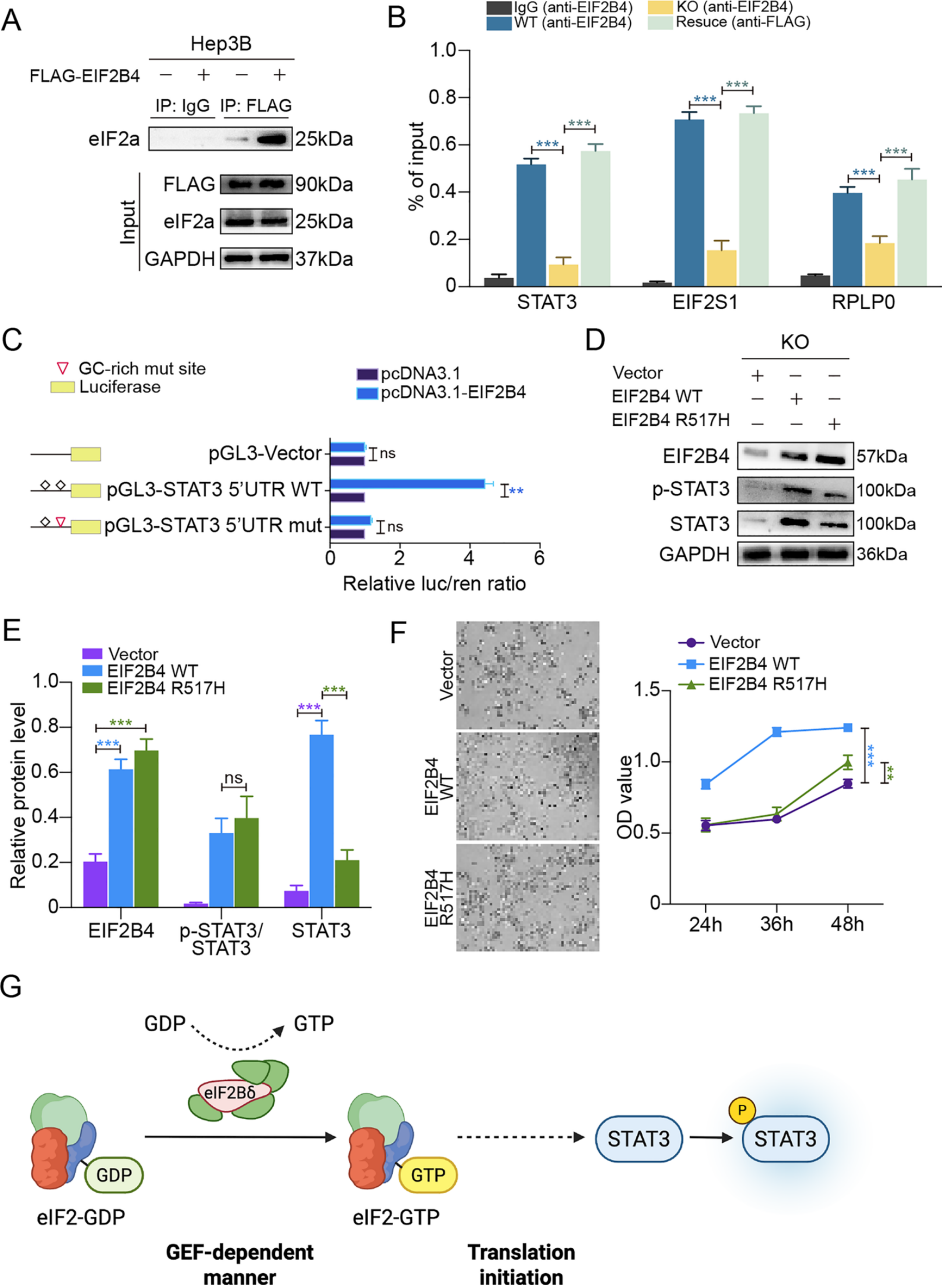
EIF2B4 Facilitates STAT3 Translation through a GEF-Dependent Mechanism and Interacts with the Translation Initiation Complex. (**A**).Co-IP showing interaction between FLAG-EIF2B4 and eIF2α in Hep3B cells. (**B**). RIP-qPCR showing EIF2B4 binding to STAT3, EIF2S1, and RPLP0 mRNAs in WT and FLAG-rescue cells, but not in EIF2B4-KO. (**C**) Dual-luciferase assay showing EIF2B4 promotes STAT3 5′UTR-mediated translation and signal reduced upon mutation of GC-rich region. (**D**) Immunoblot of STAT3 and p-STAT3 in EIF2B4-KO cells reconstituted with vector, WT, or GEF-deficient mutant (R517H) EIF2B4. (**E**) Quantification of protein levels in panel D, normalized to GAPDH. (**F**) Bright-field images and CCK8 assay showing GEF function is required for EIF2B4-induced cell proliferation. (**G**) Schematic model illustrating EIF2B4’s role in promoting STAT3 translation and downstream oncogenic signaling via the translation initiation machinery

To delineate the molecular basis of EIF2B4-mediated translational regulation of STAT3, we first investigated whether EIF2B4 physically associates with translation initiation machinery [23]. In Hep3B cells overexpressing FLAG-tagged EIF2B4, co-immunoprecipitation (Co-IP) using anti-FLAG antibody successfully pulled down eIF2α, but not in vector controls, suggesting a specific interaction between EIF2B4 and eIF2α (Fig. 7A). While input lysates confirmed comparable protein levels across samples.

To examine whether EIF2B4 binds translationally relevant mRNAs, RIP-qPCR was performed using anti-EIF2B4 antibody across four conditions: IgG control, wild-type (WT), EIF2B4 knockout (KO), and FLAG-EIF2B4 rescue. As shown in Fig. 7B, the binding of STAT3, EIF2S1, and RPLP0 mRNAs to EIF2B4 was markedly reduced in the KO group (comparable to IgG), and significantly restored by EIF2B4 rescue, even slightly exceeding WT levels, indicating EIF2B4‘s direct association with these transcripts.

To pinpoint the regulatory region responsible for STAT3 translational activation, we constructed luciferase reporters containing either wild-type STAT3 5′UTR or a high-GC segment mutant. Dual-luciferase assays revealed that EIF2B4 OE significantly enhanced reporter activity in the wild-type 5′UTR group but failed to do so upon GC-rich segment mutation (Fig. 7C), supporting that EIF2B4 facilitates STAT3 translation via direct 5′UTR interaction.

Given EIF2B4’s canonical role as a GEF, we next investigated whether this function is required for STAT3 translation. In EIF2B4 KO Hep3B cells, we ectopically expressed either WT or GEF-inactivating mutant (R517H) EIF2B4. Western blot analysis showed that while WT EIF2B4 restored STAT3 protein levels, the R517H mutant failed to do so, despite comparable expression of EIF2B4 (Fig. 7D, E), confirming the necessity of GEF activity in STAT3 regulation.

At the phenotypic level, we observed parallel results: EIF2B4-induced proliferation in Hep3B cells, as assessed by CCK-8 assays, was abrogated by the GEF-deficient R517H mutant (Fig. 7F), further reinforcing the functional dependency on GEF activity (quantify OD values or fold changes).

Finally, as illustrated in Fig. 7G, we propose a working model in which EIF2B4 interacts with the eIF2α translation initiation complex and binds the STAT3 5′UTR to promote its translation in a GEF-dependent manner, ultimately driving oncogenic progression and immune resistance in HCC.

## Discussion

This study systematically deciphers the multifaceted oncogenic mechanisms of EIF2B4, a core component of the eukaryotic translation initiation complex, in HCC. By integrating TCGA cohort analyses, functional experiments, and in vivo models, we provide the first clinical evidence linking elevated EIF2B4 expression to poor HCC prognosis, while mechanistically implicating its translational regulatory role in tumor progression and immune evasion. At the cell-autonomous level, EIF2B4 sustains efficient translation of STAT3 mRNA, driving STAT3 mediated cell cycle progression (via CCND1 upregulation) and apoptosis suppression (via BCL2/MCL1 induction) (Figs. 4–5). Complementing this, our in vivo models reveal that EIF2B4 fosters a “cold” tumor phenotype by impeding immune cell infiltration and upregulating PD-L1 to enforce adaptive immune resistance, thereby positioning EIF2B4 as a promising therapeutic target to enhance ICB sensitivity.

The role of translation initiation factors in cancer has garnered increasing attention, yet prior studies predominantly focused on upstream regulators like eIF4E or phosphorylated eIF2α [24]. For instance, eIF4E-driven selective translation of Hgf, Spp1 and Bgn suppresses immunotherapy by recruiting MDSCs in prostate cancer [25], while eIF2α phosphorylation-mediated integrated stress response (ISR) underlies sorafenib resistance of HCC [26]. In contrast, the tumor-specific role of EIF2B4, the catalytic subunit of the eIF2B complex, remained unexplored. Our work uncovers its dual oncogenic functions in HCC: intensifying malignant traits through STAT3-dependent translational reprogramming, and sculpting an immunosuppressive niche. Mechanistically, we further demonstrated that EIF2B4 promotes STAT3 translation in a GEF-dependent manner by interacting with the eIF2 complex, and this effect is mediated through its recognition of the 5′UTR of STAT3 mRNA. These findings align EIF2B4 not only as a translational modulator but also as a structural component of the translation initiation machinery, distinct from canonical cap-binding regulators like eIF4E [23, 27]. Crucially, EIF2B4’s restricted expression in normal tissues suggests it may represent a selective translational vulnerability with potentially lower off-target toxicity than broader translation initiation inhibitors.

STAT3 emerges as the central effector of EIF2B4’s pleiotropic effects. Despite compensatory upregulation of STAT3 mRNA upon EIF2B4 knockout, STAT3 protein levels plummet, revealing an irreplaceable reliance on translational efficiency over transcriptional compensation—a phenomenon mirroring the “bottleneck” effect observed in KRAS-mutant tumors where c-MYC translation dictates oncogenic output [28]. This translational dominance underscores EIF2B4’s role as a critical vulnerability in HCC.

While our findings illuminate EIF2B4’s molecular network, certain limitations warrant consideration: The resolution of immune infiltration analysis based on qPCR markers (e.g., CD68, CD8A) may be confounded by tumor purity and cellular heterogeneity, which we have now complemented with targeted flow cytometry in syngeneic tumors (CD3^+^CD8^+^ T cells, CD4^+^ Foxp3^+^ Tregs, and F4/80^+^ CD206^+^ M2 macrophages). Nonetheless, comprehensive high-dimensional profiling, including CD4^+^ T cell subset resolution and explicit M1/M2 polarization assessments, together with spatial transcriptomic mapping, will be important in future work [29]. Additionally, our in vitro co-culture models, while informative, oversimplify the tumor microenvironment by excluding stromal and myeloid components. Patient-derived organoids or humanized mouse models could better recapitulate the multicellular dynamics of EIF2B4-mediated immune evasion [30–32]. Furthermore, the compensatory mechanisms driving JAK1/STAT3 transcriptional upregulation post-EIF2B4 knockout remain unresolved, warranting CRISPR-based screens to identify stress-responsive regulators such as ATF4 or NF-κB. Additionally, although our data establish that EIF2B4’s GEF activity is indispensable for STAT3 translational upregulation, the detailed biochemical mechanism remains undefined. EIF2B4 catalyzes GDP-GTP exchange on eIF2 to regenerate the eIF2–GTP–Met-tRNA^i^ ternary complex required for initiation; how this activity yields preferential enhancement of STAT3 translation-potentially via 5′UTR structural elements or cooperation with RNA-binding cofactors that guide ternary-complex loading-remains to be elucidated. Future biochemical (e.g., ribosome profiling) and structural approaches will be necessary to resolve this mechanism.

Our findings hold significant translational implications: A predictive model integrating EIF2B4 expression levels with TMB and PD-L1 status may optimize treatment stratification for hepatocellular carcinoma patients receiving immune checkpoint therapy. Furthermore, our results provide a rationale for exploring EIF2B4 as a potential therapeutic target in combination with ICB. While no specific EIF2B4 inhibitors are currently available, future drug development efforts could focus on disrupting its GEF interface or associated translational machinery to achieve high specificity with reduced systemic toxicity [33].

In conclusion, this study redefines EIF2B4 as a linchpin of translational-immune crosstalk in HCC. By bridging cell-intrinsic proliferation and microenvironmental immunosuppression, our work provides a framework for novel combination therapies and underscores the urgency of advancing EIF2B4-targeted strategies into clinical trials to address unmet needs in HCC treatment.

## Electronic supplementary material

Below is the link to the electronic supplementary material.

Supplementary Material 1

## Data Availability

The pan-cancer and HCC sequencing datasets analyzed in this study were obtained from The Cancer Genome Atlas (TCGA) database (https://portal.gdc.cancer.gov/). Other data supporting the findings of this study are available from the corresponding author upon reasonable request.

## Abbreviations

HCC: Hepatocellular carcinoma
GEF: Guanine nucleotide exchange factor
UTR: Untranslated region
RIP: RNA immunoprecipitation
LIHC: Liver Hepatocellular Carcinoma
ICB: Immune Checkpoint Blockade
TME: Tumor Microenvironment
eIF2B: Eukaryotic translation initiation factor 2B
EIF2B4: Eukaryotic Translation Initiation Factor 2B Subunit Delta
TPM: Transcripts Per Million
DEGs: Differentially Expressed Genes
GO: Gene Ontology
KM: Kaplan-Meier
ROC: Receiver Operating Characteristic
TMB: Tumor Mutation Burden
KO: Knock Out
OS: Overall Survival
AUC: Area Under the Curve
ISR: Integrated Stress Response
p-STAT3: phosphorylated STAT3

## References

[1] Z.J. Brown, D.I. Tsilimigras, S.M. Ruff, A. Mohseni, I.R. Kamel, J.M. Cloyd et al., Management of hepatocellular carcinoma: a review. JAMA Surg. **158**(4), 410–420 (2023) 36790767 10.1001/jamasurg.2022.7989

[2] A.G. Singal, F. Kanwal, J.M. Llovet, Global trends in hepatocellular carcinoma epidemiology: implications for screening, prevention and therapy. Nat Rev Clin Oncol. **20**(12), 864–884 (2023) 37884736 10.1038/s41571-023-00825-3

[3] F. Foerster, S.J. Gairing, S.I. Ilyas, P.R. Galle, Emerging immunotherapy for HCC: a guide for hepatologists. Hepatology **75**(6), 1604–1626 (2022) 35253934 10.1002/hep.32447 PMC9117522

[4] R.S. Finn, S. Qin, M. Ikeda, P.R. Galle, M. Ducreux, T.-Y. Kim et al., Atezolizumab plus bevacizumab in unresectable hepatocellular carcinoma. NEJM. EviD. **382**(20), 1894–1905 (2020) 10.1056/NEJMoa1915745 32402160

[5] A.B. El-Khoueiry, B. Sangro, T. Yau, T.S. Crocenzi, M. Kudo, C. Hsu et al., Nivolumab in patients with advanced hepatocellular carcinoma (CheckMate 040): an open-label, non-comparative, phase 1/2 dose escalation and expansion trial. Lancet **389**(10088), 2492–2502 (2017) 28434648 10.1016/S0140-6736(17)31046-2 PMC7539326

[6] C. Weller, O. Bartok, C.S. McGinnis, H. Palashati, T.-G. Chang, D. Malko et al., Translation dysregulation in cancer as a source for targetable antigens. Cancer Cell. (2025) 10.1016/j.ccell.2025.03.003 PMC12074880 40154482

[7] L. He, X. Zhang, F. Shi, H. Zhang, Y. Chen, K. Sun et al., Reprograming immunosuppressive microenvironment by eIF4G1 targeting to eradicate pancreatic ductal adenocarcinoma. Cell. Rep. Med. **5**(10) (2024) 10.1016/j.xcrm.2024.101731 PMC11513812 39303711

[8] W. Zhang, Y. Sun, L. Bai, L. Zhi, Y. Yang, Q. Zhao et al., RBMS1 regulates lung cancer ferroptosis through translational control of SLC7A11. J. Clin. Invest. **131**(22) (2021) 10.1172/JCI152067 PMC8592553 34609966

[9] T. Ito, J.D. Wuerth, F. Weber, Protection of eIF2B from inhibitory phosphorylated eIF2: a viral strategy to maintain mRNA translation during the PKR-triggered integrated stress response. J. Biol. Chem. **299**(11), 105287 (2023) 37742919 10.1016/j.jbc.2023.105287 PMC10616414

[10] A.F. Zyryanova, K. Kashiwagi, C. Rato, H.P. Harding, A. Crespillo-Casado, L.A. Perera et al., ISRIB blunts the integrated stress response by allosterically antagonising the inhibitory effect of phosphorylated eIF2 on eIF2B. Mol. Cell. **81**(1), 88–103. e6 (2021) 33220178 10.1016/j.molcel.2020.10.031 PMC7837216

[11] C. Sidrauski, J.C. Tsai, M. Kampmann, B.R. Hearn, P. Vedantham, P. Jaishankar et al., Pharmacological dimerization and activation of the exchange factor eIF2B antagonizes the integrated stress response. Elife **4**, e07314 (2015) 25875391 10.7554/eLife.07314 PMC4426669

[12] Z. Ren, J. Zhang, D. Zheng, Y. Luo, Z. Song, F. Chen et al., Identification of prognosis-related oxidative stress model with immunosuppression in HCC. Biomedicines **11**(3), 695 (2023) 36979675 10.3390/biomedicines11030695 PMC10045103

[13] T. Zhang, H. Zheng, D. Lu, G. Guan, D. Li, J. Zhang et al., RNA binding protein TIAR modulates HBV replication by tipping the balance of pgRNA translation. Signal. Transduct. Target. Ther. **8**(1), 346 (2023) 37699883 10.1038/s41392-023-01573-7 PMC10497612

[14] N. Malik, H. Yan, N. Moshkovich, M. Palangat, H. Yang, V. Sanchez et al., The transcription factor CBFB suppresses breast cancer through orchestrating translation and transcription. Nat. Commun. **10**(1), 2071 (2019) 31061501 10.1038/s41467-019-10102-6 PMC6502810

[15] P.-P. Hou, L.-J. Luo, H.-Z. Chen, Q.-T. Chen, X.-L. Bian, S.-F. Wu et al., Ectosomal PKM2 promotes HCC by inducing macrophage differentiation and remodeling the tumor microenvironment. Mol. Cell. **78**(6), 1192–206. e10 (2020) 32470318 10.1016/j.molcel.2020.05.004

[16] C.S. McGinnis, Z. Miao, D. Superville, W. Yao, A. Goga, N.E. Reticker-Flynn et al., The temporal progression of lung immune remodeling during breast cancer metastasis. Cancer Cell. **42**(6), 1018–31. e6 (2024) 38821060 10.1016/j.ccell.2024.05.004 PMC11255555

[17] Y. Liu, Z. Xun, K. Ma, S. Liang, X. Li, S. Zhou et al., Identification of a tumour immune barrier in the HCC microenvironment that determines the efficacy of immunotherapy. J. Hepatol. **78**(4), 770–782 (2023) 36708811 10.1016/j.jhep.2023.01.011

[18] K. Yoshihara, M. Shahmoradgoli, E. Martínez, R. Vegesna, H. Kim, W. Torres-Garcia et al., Inferring tumour purity and stromal and immune cell admixture from expression data. Nat. Commun. **4**(1), 2612 (2013) 24113773 10.1038/ncomms3612 PMC3826632

[19] J. Budczies, D. Kazdal, M. Menzel, S. Beck, K. Kluck, C. Altbürger et al., Tumour mutational burden: clinical utility, challenges and emerging improvements. Nat Rev Clin Oncol. **21**(10), 725–742 (2024) 39192001 10.1038/s41571-024-00932-9

[20] D.-C. Chiu, V.-H. Yuen, J.-S. Cheu, L.L. Wei, V. Ting, M. Fehlings et al., Hepatocellular carcinoma cells up-regulate PVRL1, stabilizing PVR and inhibiting the cytotoxic T-cell response via TIGIT to mediate tumor resistance to PD1 inhibitors in mice. Gastroenterology **159**(2), 609–623 (2020) 32275969 10.1053/j.gastro.2020.03.074

[21] W.-M. Hu, M. Li, J.-Z. Ning, Y.-Q. Tang, T.-B. Song, L.-Z. Li et al., FAM171B stabilizes vimentin and enhances CCL2-mediated TAM infiltration to promote bladder cancer progression. J. Exp. Clin. Cancer Res. **42**(1), 290 (2023) 37915048 10.1186/s13046-023-02860-5 PMC10621219

[22] S. Di, C. Fan, Z. Ma, M. Li, K. Guo, D. Han et al., PERK/eIF-2α/CHOP pathway dependent ROS generation mediates butein-induced non-small-cell lung cancer apoptosis and G2/M phase arrest. Int. J. Biol. Sci. **15**(8), 1637 (2019) 31360107 10.7150/ijbs.33790 PMC6643215

[23] J. Wang, X. Zhang, G.H. Greene, G. Xu, X. Dong, PABP/purine-rich motif as an initiation module for cap-independent translation in pattern-triggered immunity. Cell **185**(17), 3186–200. e17 (2022) 35907403 10.1016/j.cell.2022.06.037 PMC9391319

[24] W.C. Merrick, eIF4F: a retrospective. J. Biol. Chem. **290**(40), 24091–24099 (2015) 26324716 10.1074/jbc.R115.675280 PMC4591800

[25] D. Brina, A. Ponzoni, M. Troiani, B. Calì, E. Pasquini, G. Attanasio et al., The Akt/mTOR and MNK/eIF4E pathways rewire the prostate cancer translatome to secrete HGF, SPP1 and BGN and recruit suppressive myeloid cells. Nat. Cancer. **4**(8), 1102–1121 (2023) 37460872 10.1038/s43018-023-00594-z PMC11331482

[26] H. Dai, B. Wu, Y. Ge, Y. Hao, L. Zhou, R. Hong et al., Deubiquitylase OTUD3 regulates integrated stress response to suppress progression and sorafenib resistance of liver cancer. Cell. Rep. **43**(7. 2024) 10.1016/j.celrep.2024.114487 38996071

[27] B.-S. Shin, J.-R. Kim, S.E. Walker, J. Dong, J.R. Lorsch, T.E. Dever, Initiation factor eIf2γ promotes eIF2-GTP-Met-tRnaimet ternary complex binding to the 40S ribosome. Nat. Struct. Mol. Biol. **18**(11), 1227–1234 (2011) 22002225 10.1038/nsmb.2133 PMC3210414

[28] S. Casacuberta-Serra, Í. González-Larreategui, D. Capitán-Leo, L. Soucek, MYC and KRAS cooperation: from historical challenges to therapeutic opportunities in cancer. Signal. Transduct. Target. Ther. **9**(1), 205 (2024) 39164274 10.1038/s41392-024-01907-z PMC11336233

[29] W. Chen, M. Chen, L. Hong, A. Xiahenazi, M. Huang, N. Tang et al., M2-like tumor-associated macrophage-secreted CCL2 facilitates gallbladder cancer stemness and metastasis. Exp Hematol Oncol. **13**(1), 83 (2024) 39138521 10.1186/s40164-024-00550-2 PMC11320879

[30] J.T. Neal, X. Li, J. Zhu, V. Giangarra, C.L. Grzeskowiak, J. Ju et al., Organoid modeling of the tumor immune microenvironment. Cell **175**(7), 1972–88. e16 (2018) 30550791 10.1016/j.cell.2018.11.021 PMC6656687

[31] Z. Zou, Z. Lin, C. Wu, J. Tan, J. Zhang, Y. Peng et al., Micro-engineered organoid-on-a-chip based on mesenchymal stromal cells to predict immunotherapy responses of HCC patients. Adv. Sci. **10**(27), 2302640 (2023) 10.1002/advs.202302640 PMC10520686 37485650

[32] R. Xu, X. Zhou, S. Wang, C. Trinkle, Tumor organoid models in precision medicine and investigating cancer-stromal interactions. Pharmacol. Ther. **218**, 107668 (2021) 32853629 10.1016/j.pharmthera.2020.107668 PMC7855432

[33] Y. Sekine, A. Zyryanova, A. Crespillo-Casado, P.M. Fischer, H.P. Harding, D. Ron, Mutations in a translation initiation factor identify the target of a memory-enhancing compound. Science **348**(6238), 1027–1030 (2015) 25858979 10.1126/science.aaa6986 PMC4538794

