# Supplementary material for: EIF2B4 promotes hepatocellular carcinoma progression and immune evasion by driving STAT3 translation via a GEF-dependent mechanism
Source: Cell Oncol (Dordr). 2025 Oct 27;48(6):1919–33. doi: 10.1007/s13402-025-01117-x (PMC12698737; doi:10.1007/s13402-025-01117-x)

**Title**

EIF2B4 Promotes Hepatocellular Carcinoma Progression and Immune Evasion by Driving STAT3 Translation via a GEF-Dependent Mechanism

**Journal**

*Cellular Oncology*

**Authors**

Yirui He^*,1^, Yunhe Li^*,2^, Yayi Chen^*,1^, Sha Liu^3^, Jia Liu^1^, Rui Wei^1^, Jiapeng Zhang^#,1^

**Affiliation**

1. Department of Hematology, Department of Urology and Institute of Urology, Department of Pulmonary and Critical Care Medicine, West China Hospital, Sichuan University, Chengdu, China.

2. Department of Thoracic and Cardiac Surgery, Second Affiliated Hospital of Chongqing Medical University, Chongqing, China.

3. Department of Geriatrics, Sichuan Second Hospital of Traditional Chinese Medicine, Chengdu, China.

*These authors contributed equally to this work and should be considered co-first authors

# Corresponding author

Email: [jp_zhang@scu.edu.cn](mailto:jp_zhang@scu.edu.cn)

Supplementary material

Supplementary figure


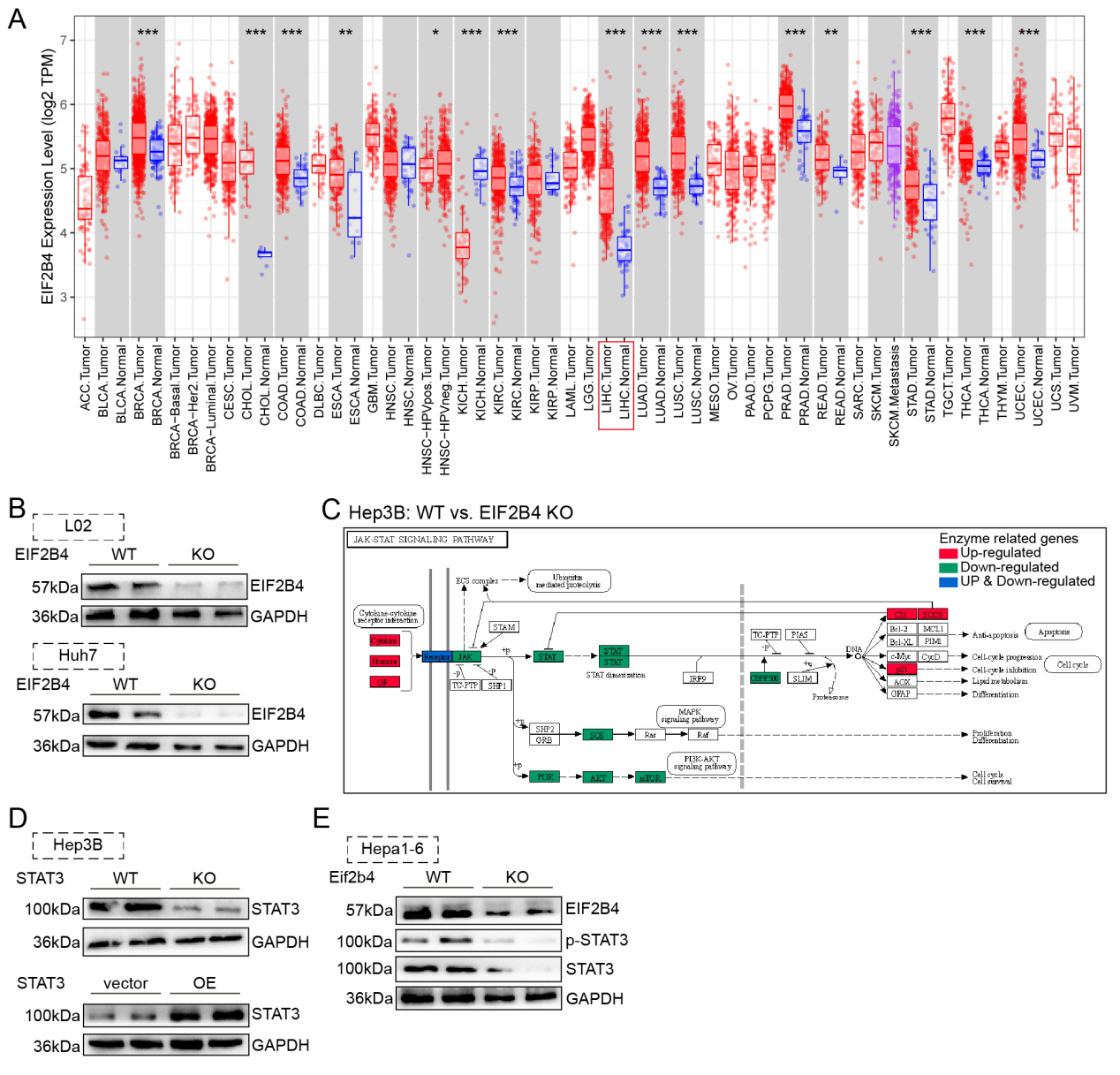


1. Differential expression of EIF2B4 between tumor and adjacent normal tissues across various cancer types in the TCGA database.
2. Representative immunoblot showing validation of EIF2B4 knockout in cell lines.
3. Transcriptomic changes of key genes in the JAK-STAT signaling pathway before and after EIF2B4 knockout in Hep3B cells.
4. Representative immunoblot showing validation of EIF2B4 overexpression in Hep3B.
5. Representative immunoblot showing validation of EIF2B4 konckout in Hepa1-6.

Uncropped immunoblots

Figure5


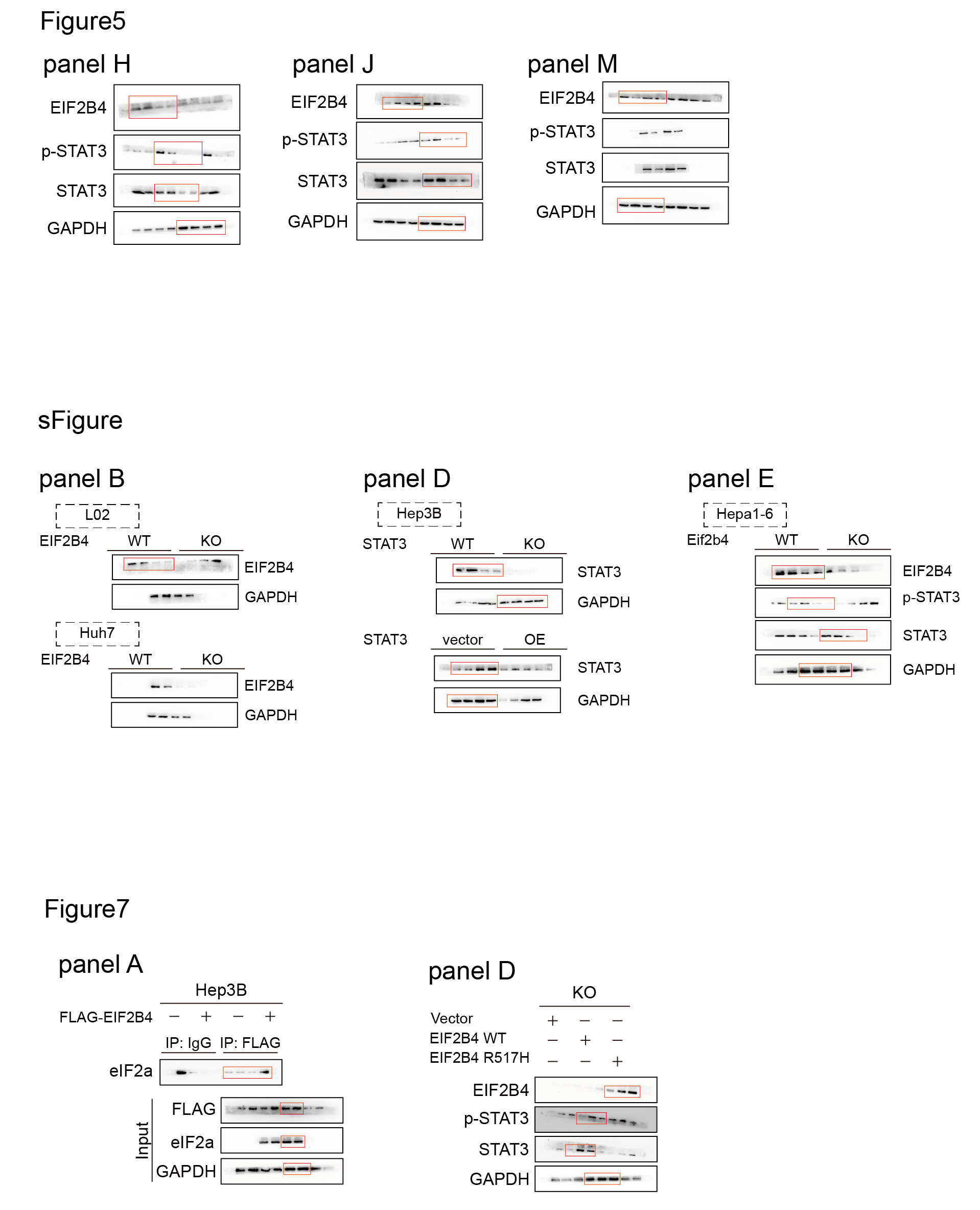


Figure7


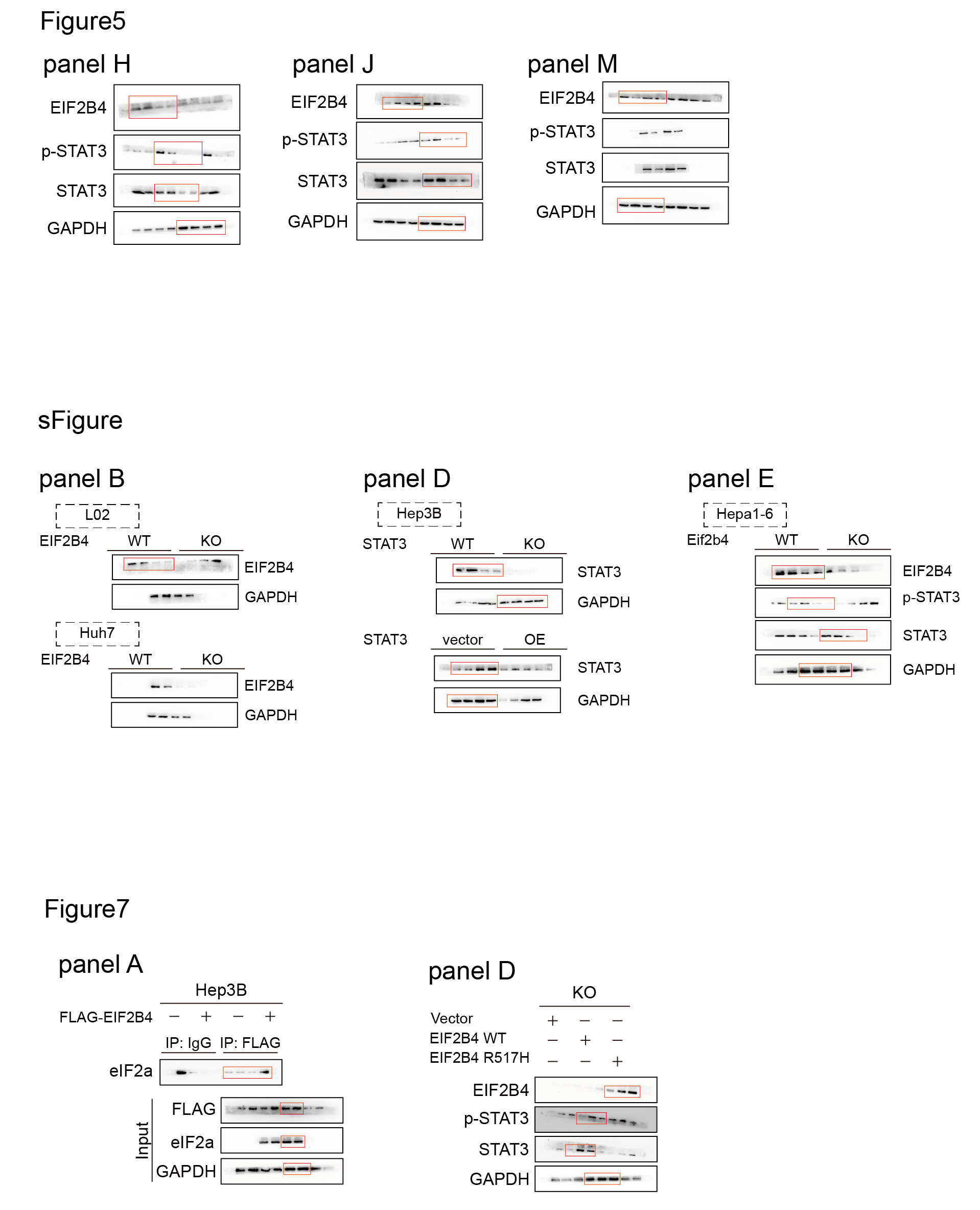


Supplementary figure


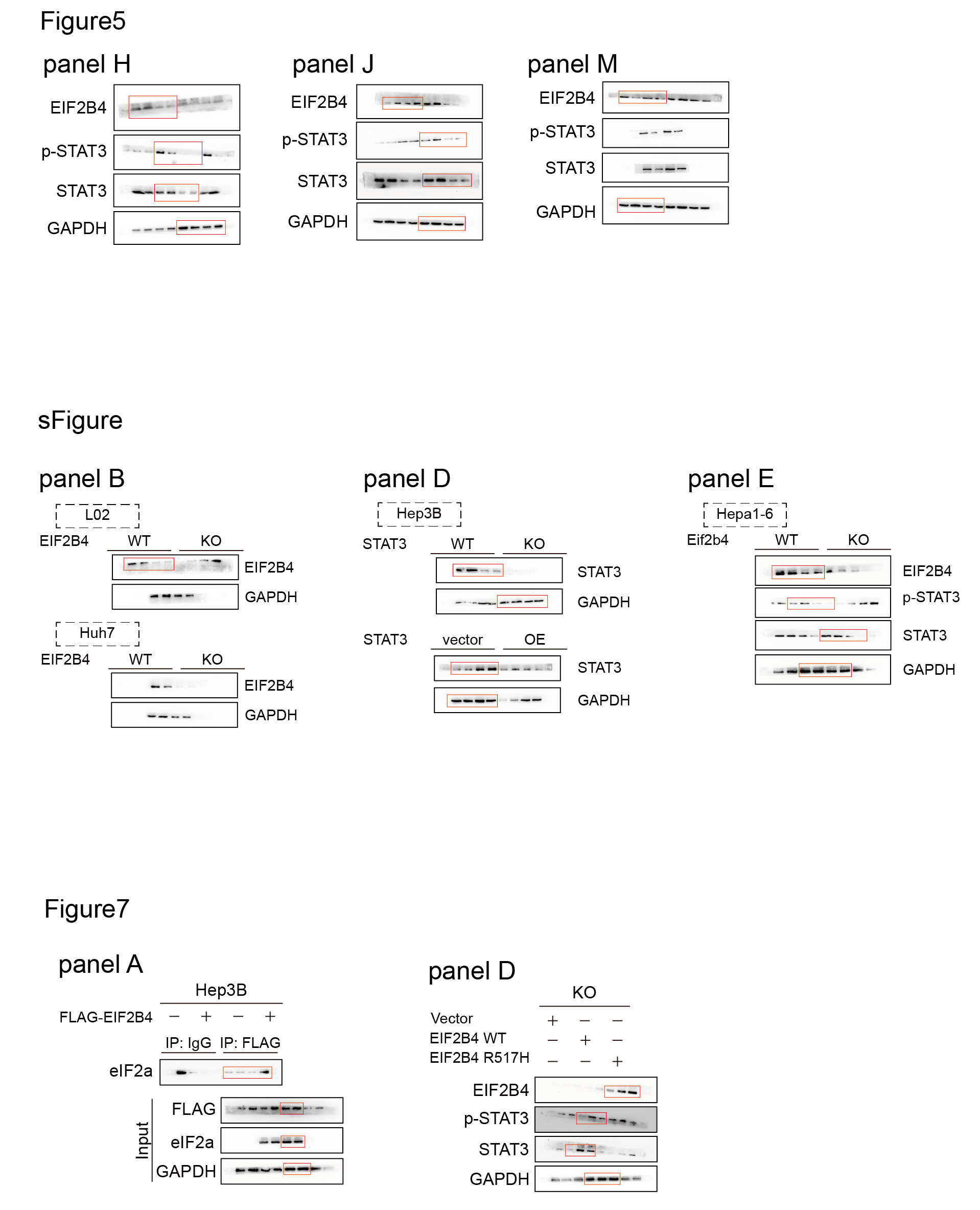

Supplement: Supplementary file 1 — Supplementary Material 1 [file 13402_2025_1117_MOESM1_ESM.docx]
